# Identification and characteristics of differentially expressed genes under UV-B stress in *Gossypium hirsutum*

**DOI:** 10.3389/fpls.2024.1529912

**Published:** 2025-01-15

**Authors:** Xiaolin Song, Yingjie Zhu, Ying Bao

**Affiliations:** School of Life Sciences, Qufu Normal University, Qufu, Shandong, China

**Keywords:** photosynthesis, UV-B stress, phenylpropanoid metabolism, Gossypium hirsutum, co-expression network, anthocyanin biosynthesis

## Abstract

**Objective:**

This study aimed to screen the differentially expressed genes (DEGs) of *Gossypium hirsutum* under UV-B stress and identify the significant pathways based on gene enrichment analysis results.

**Methods:**

In this study, the allotetraploid crop *G. hirsutum* was used to examine changes in various physiological indexes under UV-B stress, and screened out all DEGs under UV-B stress (16 kJ m^-2^ d^-1^) based on six leaf transcriptomes. The main enrichment pathways of DEGs were analyzed according to gene annotation. Finally, we predicted the regulatory genes of phenylpropanoid pathway under UV-B stress by co-expression network analysis, and selected *GhMYB4* for verification.

**Results:**

Gene annotation analysis revealed that DEGs were predominantly enriched in pathways related to photosynthesis and secondary metabolism. Further analysis revealed that UV-B stress impaired photosynthesis mainly by damaging photosystem II (PSII) and inhibiting electron transport, whereas *G. hirsutum* responded to UV-B stress by synthesizing secondary metabolites such as anthocyanins and lignin. We selected the regulatory genes *GhMYB4* for verification. It was found to be an anthocyanin negative regulator in response to UV-B stress.

**Conclusions:**

UV-B stress impaired photosynthesis mainly by damaging photosystem II (PSII) and inhibiting electron transport, whereas *G. hirsutum* responded to UV-B stress by synthesizing secondary metabolites such as anthocyanins and lignin.

## 1 Introduction

Ongoing changes in global climate and the stratospheric ozone layer have resulted in changes in temperature, solar ultraviolet radiation, and other factors (Bornman et al., 2020). Based on the wavelength range, UV (ultraviolet) light is divided into three types: UV-A radiations (from 315 to 400 nm), UV-B (from 280 to 315 nm), and UV-C (from 100 to 280 nm) (Iasnogorodskii, 2005). The stratospheric ozone layer can absorb the entire UV-C radiation, most UV-B radiation, and a small portion of UV-A radiation. Although only a small portion of UV-B radiation reaches the Earth’s surface, it can significantly affects plants owing to its high energy (Vanhaelewyn et al., 2020).

Low-level UV-B radiation promotes plant photomorphogenesis and adaptation. However, high-levels of UV-B radiation affect the growth of terrestrial plants, causing DNA damage, reactive oxygen species (ROS) accumulation, and photosynthetic impairment. High-level UV-B radiation, known as UV-B stress, can damage plants and lead to abnormal growth and development. Plant growth, physiological characteristics, and secondary metabolism undergo significant changes under UV-B stress. Excessive UV-B radiation can lead to plant cell death, and its physiological effects include leaf wilting, yellowing, and bleaching (Jenkins, 2009). During photosynthesis, UV-B radiation directly damages photosystem II (PSII) by degrading the PSII proteins D1 and D2 and damaging the photosynthetic machinery (Zhou et al., 2024). In addition to photosystem damage, UV-B can promote chlorophyll degradation and reduce chlorophyll content, resulting in low photosynthetic capacity of plants (Rahimzadeh Karvansara and Razavi, 2019). UV-B exposure inhibits primary root elongation by reducing cell proliferation in the meristematic zone of Arabidopsis (Sheridan et al., 2022). Plant height, tiller number, and root length decreases in both indica and japonica rice plants under UV-B stress (Mathur et al., 2024). Seed germination and seedling development in Anatolian black pines decrease with increasing exposure time to UV radiation (Ozel et al., 2021). UV-B radiation can cause DNA and protein damage at the molecular level. UV-B stress induces DNA damage by producing cyclobutene pyrimidine dimers (CPDs) and pyrimidine (6-4) and pyrimidinone photoproducts (6-4 PPs), that affect DNA replication and transcription, thereby inhibiting plant development and metabolism (Chen et al., 2022). In addition to plant morphology, UV-B radiation also has a significant effect on plant metabolism. Phenylpropanoids, including flavonoids and polyphenols, accumulate in the epidermal cells to protect plants from potentially harmful UV-B radiation. UV-B radiation promotes flavonoid and phenol production (Liu et al., 2020; Lee et al., 2021; Li et al., 2023a). Phenylpropanoid, lignin, flavonoid production, phenolic, flavonoid, antioxidant, and anthocyanin concentrations significantly increase after UV-B irradiation (Lee et al., 2021; Gong et al., 2024). The phenylpropanoid biosynthesis pathway is the general pathway for producing compounds such as lignin, flavonoids, and anthocyanins. The phenylpropanoid pathway has two metabolic branches, flavonoid and lignin biosynthesis, that play a role in the stress response (Li et al., 2023a). UV-B stress promoted lignin accumulation, which can alleviate the damage caused by UV-B stress to *Rhododendron chrysanthum* (Gong et al., 2024). Morphological dissection revealed that the thickness of quinoa epidermal cell wall increased under UV-B stress due to the accumulation of lignin (Hilal et al., 2004). Lignin deposition in epidermal tissues of quinoa is a resistance mechanism against UV-B radiation. The carboxymethyl cellulose-lignin composite film from oil palm empty fruit bunch prepared by utilizing lignin resistance has excellent ultraviolet resistance (Haqiqi et al., 2021). After UV-B treatment, the anthocyanin content in the leaves of *Lycium ruthenicum* increased significantly, and the expression of 24 structural genes in anthocyanin synthesis pathway was up-regulated (Chen et al., 2024). Exposure to UV-B increased anthocyanin and flavone levels in the cotyledons of *Fagopyrum esculentum*, and inhibited hypocotyl elongation (Dębski et al., 2016). Higher resistance to UV-B radiation in *F. esculentum* may be attributed to its higher anthocyanin content. Plants commonly resist abiotic stress, and the effects of abiotic stress on plants are complex and involve physiological, biochemical, and molecular pathways. Therefore, a comprehensive understanding of these pathways and their interactions is essential for understanding plant stress resistance.

*AtMYB4* is a key regulator of phenylpropanoid pathway gene expression, and is the first example of a MYB protein that functions as a transcriptional repressor (Hemm et al., 2001). *AtMYB4* is one of the first proteins to transcriptionally regulate general phenylpropanoid pathway genes at the physiological level as a transcriptional repressor. It has shown that Arabidopsis MYB (*AtMYB4*) regulates the accumulation of the UV-protective compound sinapoylmalate by inhibiting the transcription of the gene encoding the phenylpropyl enzyme cinnamate 4-hydroxylase (C4H) (Hemm et al., 2001). The conclusion was verified in Arabidopsis *AtMYB4* mutant. The *AtMYB4* mutant line is more tolerant of UV-B irradiation than wild type (Jin, 2000). The increased accumulation of sinapate ester in the mutants was associated with enhanced expression of the gene encoding C4H, thereby increasing the resistance of the mutant (Jin, 2000). The defect in the mutants blocked the conversion of ferulate to L-hydroxyferulate in the general phenylpropanoid pathway (Chapple et al., 1992). As a result, the lignin of the mutant lacks the sinapic acid-derived components. *SlMYB7* inhibited anthocyanin accumulation and reduced expression of anthocyanin synthase genes in *Solanum lycopersicum* fruits (Zhang et al., 2023). Sequence similarity comparison revealed that *AtMYB4* and *SlMYB7* are homologous genes of *G. hirsutum GhMYB4* in *Arabidopsis thaliana* and *Solanum lycopersicum*. The transcription factors (TFs) *VcMYBPA1*, *MYBPA2.1*, *MYB114*, *MYBA2*, *MYBF*, and *MYB102* from blueberry (*Vaccinium corymbosum*) are activators, whereas *MYB20*, *VcMYB14*, *MYB44*, and *VcMYB4a* are inhibitors of the flavonoid biosynthetic pathway (Song et al., 2022). *BrMYB4* from turnip (*Brassica rapa*), as a negative transcriptional regulator of *BrC4H*, mediates UV-B-dependent phenylpropanoid biosynthesis (Zhang et al., 2014). The anthocyanin biosynthesis process is regulated by the MYB-bHLH-WD40 regulatory complexes at the transcription level (Schaart et al., 2012), and the R2R3-MYB subfamily is considered to be the main *MYB* regulator involved in this pathway. In our previous study, we explored and characterized the potential *MYB* regulatory genes in the anthocyanin biosynthesis pathway in *G. hirsutum* by co-expression regulatory network analysis (Zhu and Bao, 2021). It was found that *MYB*s are involved in the regulation of structural genes and multiple reaction processes of anthocyanin biosynthesis pathway.

With changes in stratospheric ozone and climate over the past few years, crops, including *G. hirsutum*, face an increasingly pronounced threat from UV-B radiation. *G. hirsutum*, produced by hybridizing two diploid species with AA and DD genomes, is an important fiber crop (Mansoor and Paterson, 2012). Plant leaves, the primary photosynthetic and transpiration organs, determine radiation interception and play a crucial role in the exchange of heat, water, and gas between plants and the external environment (Taleisnik et al., 2009). This process converts light energy and inorganic nutrients into chemical energy and organic compounds, that serve as cellular components supporting overall plant growth. Due to the large surface area, leaves are particularly vulnerable to biotic and abiotic stresses (Taleisnik et al., 2009). For instance, salinity reduces leaf expansion by affecting cell division and elongation (Taleisnik et al., 2009). Histological studies on *Vitis vinifera* leaves revealed increased leaf thickness and disordered thylakoid structures under heat stress (Salem-Fnayou et al., 2010). Additionally, water stress decreased stomatal density, size, and aperture in V*. vinifera* leaves (Ju et al., 2018). In this study, we used *G. hirsutum* as the experimental material to elucidate gene expression changes and related metabolic pathways under UV-B stress. These analyses provide more insight into the molecular mechanisms underlying the adaptation of polyploid plants to environmental changes. We identified differentially expressed genes (DEGs) and analyzed the associated metabolic pathways in *G. hirsutum* under UV-B stress through transcriptome analysis. The results offer insights for future research on plant responses to UV-B stress and the selection of tolerant crop varieties.

## 2 Materials and methods

### 2.1 Plant materials and growth conditions

From sowing to three-leaf stage, *G. hirsutum* cv. Acala Maxxa plants were cultivated in greenhouse with a long-day (LD, 16 h light/8 h dark) photoperiod at temperature of 23 ± 1°C in Qufu Normal University, China. Seeds were sown in plastic pots (10×10 cm) containing a mixture of nutrient soil and vermiculite in a ratio of 3:1, with two seeds per pot.

UV-B does simulated 30% depletion of stratospheric ozone (16 kJ m^-2^ d^-1^) (Kakani, 2003). To examine the UV-B stress response of the cotton seedlings, each of six plants were placed under normal condition (CK) or 6 h of UV-B radiation (UV-B, 16 kJ m^-2^ d^-1^), respectively. The UV-B lamp (Beijing Zhongyi Boteng Technology Co., LTD) was used to generate UV-B radiation. After treatment, the leaves from control and treated plants were collected for all experiments. All the treatments were conducted in triplicates.

The seeds of *Arabidopsis thaliana* ecotype Columbia (Col-0) were surface sterilized in 10% (v/v) sodium hypochlorite for 20 mins, washed three times with sterilized water, and then grown on medium (pH=5.8) after one day vernalization in darkness at 4°C, and then transferred to long-day (16 h light–8 h dark) growth conditions at 22°C.

### 2.2 Extraction and quantification of chlorophylls and anthocyanins

Chlorophylls were extracted with 80% (v/v) acetone (Porra et al., 1989). Two leaf disks of 1cm diameter were soaked in 2ml dimethyl sulfoxide (DMSO) at 65 °C in the dark for one hour until all leaves turned white. After cooling to room temperature, 8mL 80% (v/v) acetone was added to extract for 30 mins, and then both extracts (CK and UV-B groups) were analyzed by UV-visible scanning spectrophotometry (UV-5500, METASH, Shanghai, China) at 646.6 and 663.6 nm.

Anthocyanins were extracted with a methanol–HCl method (Kuhnen et al., 2011). The 2 g Maxxa leaves or 0.1g *Arabidopsis* leaves were soaked in 5 ml or 2ml 1% (v/v) methanol–HCL and extracted overnight at 24°C in darkness. Both extracts (CK and UV-B groups) were analyzed by UV–visible scanning spectrophotometry (UV-5500, METASH, Shanghai, China) at 530, 620, and 650 nm. All measurements were done in triplicate.

### 2.3 RNA extraction and transcriptome sequencing

Total RNAs extracted from leves of control and UV-B treatment by RNAprep Pure Plant Kits (Cat. No 4992237, TIANGEN, Beijing, China) following the manufacturer’s instructions, respectively. Purity and integrity of RNA were assessed using the RNANano 6000Assay Kit of the Bioanalyzer 2100 system (Agilent Technologies, CA, USA). Then, mRNA was purified from total RNA by using poly-T oligo-attached magnetic beads. The cDNA was synthesized through reverse transcription using random hexamer primer. Then the PCR product was purified by AMPure XP beads (BeckmanCoulter, Beverly, USA), and the library was finally obtained. After the library was qualified, six cDNA were sequenced by the Illumina NovaSeq 6000. Library construction and transcriptome sequencing were achieved by Novogene Bioinformatics Technology Co. Ltd (Novogene Co., Ltd., Beijing).

### 2.4 Gene annotation and DEG analysis

Clean data (clean reads) were obtained by removing reads containing adapter, reads containing Nbase and low quality reads from raw data using fastp (v. 0.19.7) software (Chen et al., 2018). All data were uploaded to NCBI (https://www.ncbi.nlm.nih.gov/) under the accession number PRJNA893188. Paired-end clean reads were aligned to the cotton reference genome (UTX_v2.1) using Hisat2 (v2.0.5) (Mortazavi et al., 2008). The featureCounts v1.5.0-p3 (Liao et al., 2014) was used to count the reads numbers mapped to each gene. And then FPKM (Fragments Per Kilobase of transcript per Million reads mapped) that represented the gene expression value of RNA-seq in subsequent analysis was calculated based on the length of the gene and reads count mapped to this gene.

Differential expression analysis of UV-B and CK groups (three biological replicates per condition) was performed using the DESeq2 R package (1.20.0) (Love et al., 2014). The padj ≤ 0.05 and |log2^(foldchange)^| ≥ 1 were set as the threshold for significantly differential expression genes.

### 2.5 Functional annotation and classification

The protein sequence file (Ghirsutum_527_v2.1.protein.fa) was downloaded from the cottongen website (https://www.cottongen.org/), and then submitted to the Mercator 4 website (https://www.plabipd.de/mercator_main.html) to obtain the MapMan mapping file. The log2FoldChange data file of DEGs obtained by comparative transcriptome analysis. The two files were submitted to the MapMan software (Thimm et al., 2004) for gene function classification and comprehensive pathway analysis of DEGs.

Kyoto Encyclopedia of genes and genomes (KEGG) enrichment analysis of DEGs was implemented by the clusterProfiler R package (3.8.1) (Yu et al., 2012). KEGG enrichment analyses used padj ≤ 0.05 as the threshold for significant enrichment. Subsequently, the top 20 most significant pathways of KEGG enrichment analysis were analyzed, respectively.

The structural genes, as target genes, submitted to the ccNET database (http://structuralbiology.cau.edu.cn/gossypium/compare.php) to search for their potential regulatory genes (You et al., 2017). Then, we downloaded all PCC and MR values of co-expression data of the structural genes. Based on the PCC and MR values, we constructed several co-expression regulatory networks to trace back to the regulatory genes in the pathway. Several co-expression networks were integrally generated based on threshold, and were visualized using Cytoscape software (v.3.8.0) (Shannon et al., 2003).

### 2.6 Real-time quantitative polymerase chain reaction analysis

Total RNA was extracted from leaves and the cDNA was synthesized using the HiScript II 1st Strand cDNA Synthesis Kit (+gDNA wiper) (Cat. No R212-01, Vazyme, Nanjing, Jiangsu Province, China), primers designed with Oligo are shown in **Supplementary Table S1**. The qRT-PCR experiment was carried out using an CFX Connect Real-time System (BIO-RAD, California, USA) and SYBR Green Premix Pro Taq HS qPCR Kit (Cat. No AG11701, Accurate Biology, Hangzhou, Zhejiang Province, China). The reaction system was as follows: 95°C for 30s, followed by 40 cycles of 95°C for 5 s, and 60°C for 30 s. Each sample was repeated three times, and gene *GhPU1* (*Gohir.A01G131900*) was used as internal control (Xiao et al., 2007). Each primer pair was validated the specifcity by melt curve analysis, and relative expression of these genes were calculated according to the 2^–ΔΔCT^ method (Schmittgen and Livak, 2008). T-test was applied for significant differences among treatments at *P* < 0.05 level. The results were shown as the means ± standard error of three replicates.

### 2.7 Functional verification

The anthocyanin biosynthesis process is considered to be regulated by the MYB-bHLH-WD40 regulatory complexes. And our previous study explored the potential *MYB* genes in anthocyanin biosynthesis in *G. hirsutum*. Therefore, we selected a *MYB* gene (*Gohir.A01G153200*, *GhMYB4*) for functional verification. The *GhMYB4* coding sequences were retrieved from CottonFGD (https://cottonfgd.org/). Genes (**Supplementary Table S2**) were amplified from the Maxxa cDNA library and the PCR products were cloned into pCAMBIA1300-GFP vector according to the instructions of the ClonExpress II One Step Cloning Kit (Cat. No C112-01/02, Vazyme, Nanjing, Jiangsu Province, China). The vector was subsequently transformed into Arabidopsis Col-0 plants via the Agrobacterium-mediated foral-dip method. The relative expression levels of *GhMYB4* and *GhC4H* genes under UV-B stress and overexpression lines were measured via qRT-PCR (**Supplementary Table S2**). Each sample was repeated three times, and gene *UBQ10* (*AT4G05320*) was used as internal control.

## 3 Results

### 3.1 UV-B treatment triggers physiological changes in *G. hirsutum*

**Figure 1:**
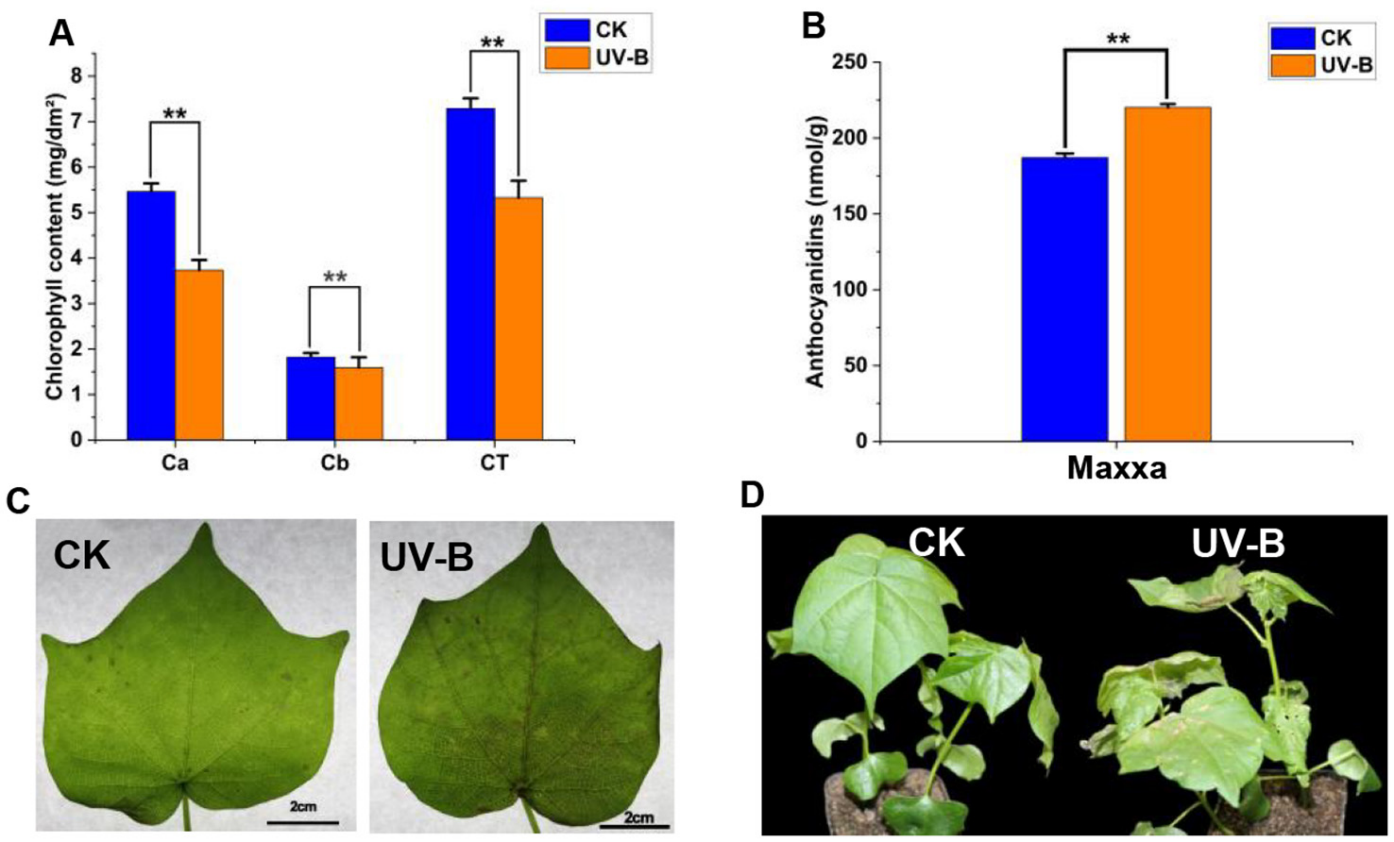
Stress treatment, and physiological index analysis of *G. hirsutum*. **(A)**, The chlorophyll content (C_a_, C_b_, C_T_) in leaves. **(B)**, The anthocyanins content in leaves. **(C, D)**, Leaves and plant phenotypes under natural condition for 6 h (CK) or UV-B (16 kJ m^-2^ d^-1^) (UV-B) for 6 h. C_a_, Chlorophyll a; C_b_, Chlorophyll b; C_T_, Total chlorophyll. **P < 0.01.

Chlorophyll and anthocyanin contents in the leaves of *G. hirsutum* were measured immediately after 6 h of UV-B treatment, and detailed physiological responses were observed. We found that C_a_, C_b_, and C_T_ contents significantly decreased following UV-B treatment, conversely anthocyanin content significantly increased (**Figures 1A, B**). Anthocyanins, among the most common stress-resistant compounds in plants, are likely synthesized by *G. hirsutum* to resist UV-B stress. After two days of UV-B treatment, the leaves exhibited visible signs of drying and damage compared to that of the control plants (**Figure 1C**). This indicates that UV-B stress (16 kJ m^-2^ d^-1^, lasting 6 h) caused severe damage to the cotton plants (**Figure 1D**).

### 3.2 Transcriptome analyses and DEGs identification of *G. hirsutum* under UV-B stress

To investigate the potential pathways and genes of cotton response to UV-B stress, RNA-seq analysis was conducted on leaves under normal growth conditions and UV-B stress (three-leaf stage, UV-B, 16 kJ m^-2^ d^-1^). Six high-quality transcriptomes were obtained (**Supplementary Table S3**). The raw reads of the libraries have been deposited in the NCBI Sequence Read Archive (SRA) database (accession number: PRJNA893188). The FPKM value was calculated for each unigene, and padj ≤ 0.05 and |log2^(foldchange)^| ≥ 1 were set as the threshold for DEGs. A total of 24,257 DEGs were detected in the CK/UV-B comparison, comprising 12,645 upregulated and 11,612 downregulated.

**Figure 2:**
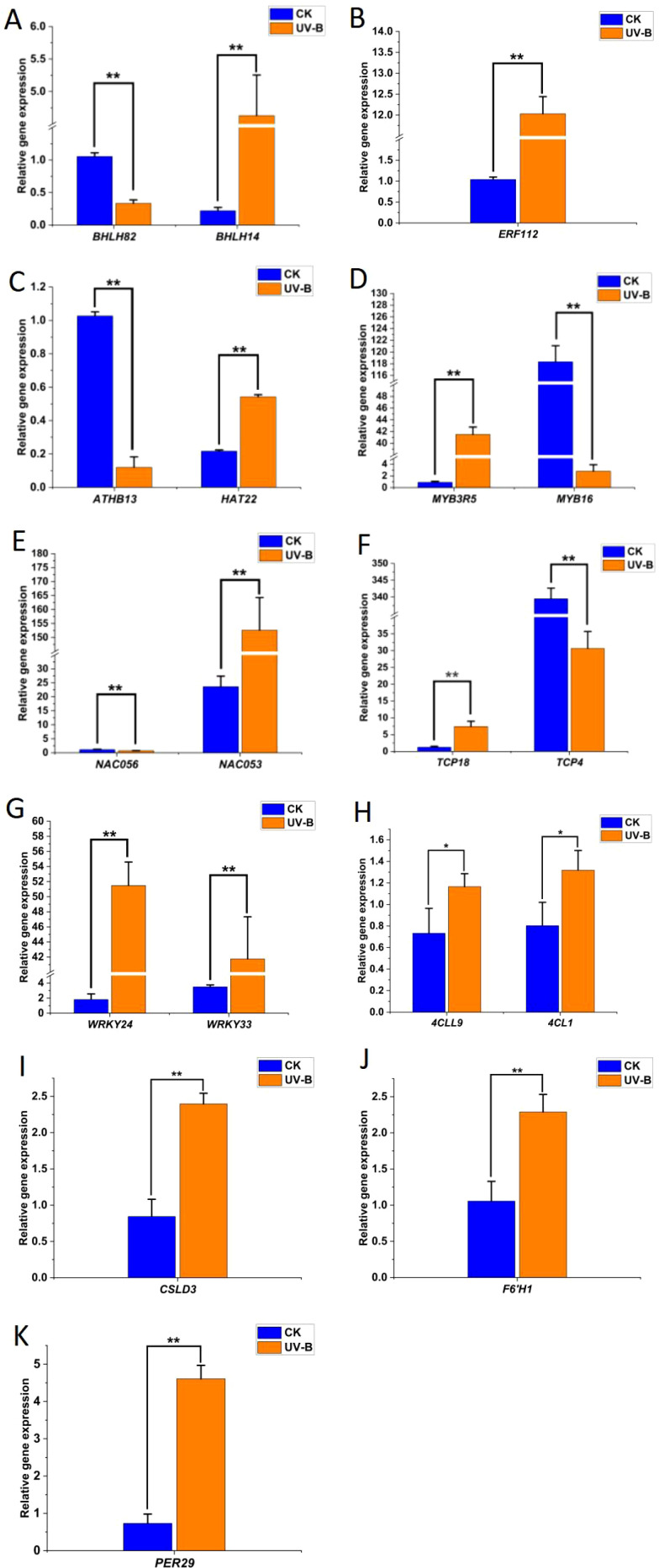
qRT-PCR analysis of expression changes of representative genes in the phenylpropanoid pathway under UV-B stress. **(A–K)**, The relative gene expression of *bHLH*, *ERF*, *HD-Zip*, *MYB*, *NAC*, *TCP*, *WRKY*, *4CL*, *CSLD*, *F6’H*, and *PER*. Statistical significance was calculated using *T*-test; *P < 0.05, **P < 0.01.

To ensure the reliability of transcriptome sequencing data, we evaluated the expression patterns of 18 randomly selected genes associated with UV-B stress response using qRT-PCR. These genes showed different functions and were involved in a variety of pathways, including four genes (*4CL1*, *4CLL9*, *F6’H1*, and *PER29*) involved in the phenylpropanoid biosynthesis pathway, one gene (*CSLG3)* involved in cellulose synthesis and 13 TFs (**Figure 2**). The expression trends of these genes under UV-B stress were highly consistent with the RNA-seq results, confirming the reliability of RNA sequencing.

### 3.3 MapMan analysis of DEGs based on signaling and metabolic pathways

**Figure 3:**
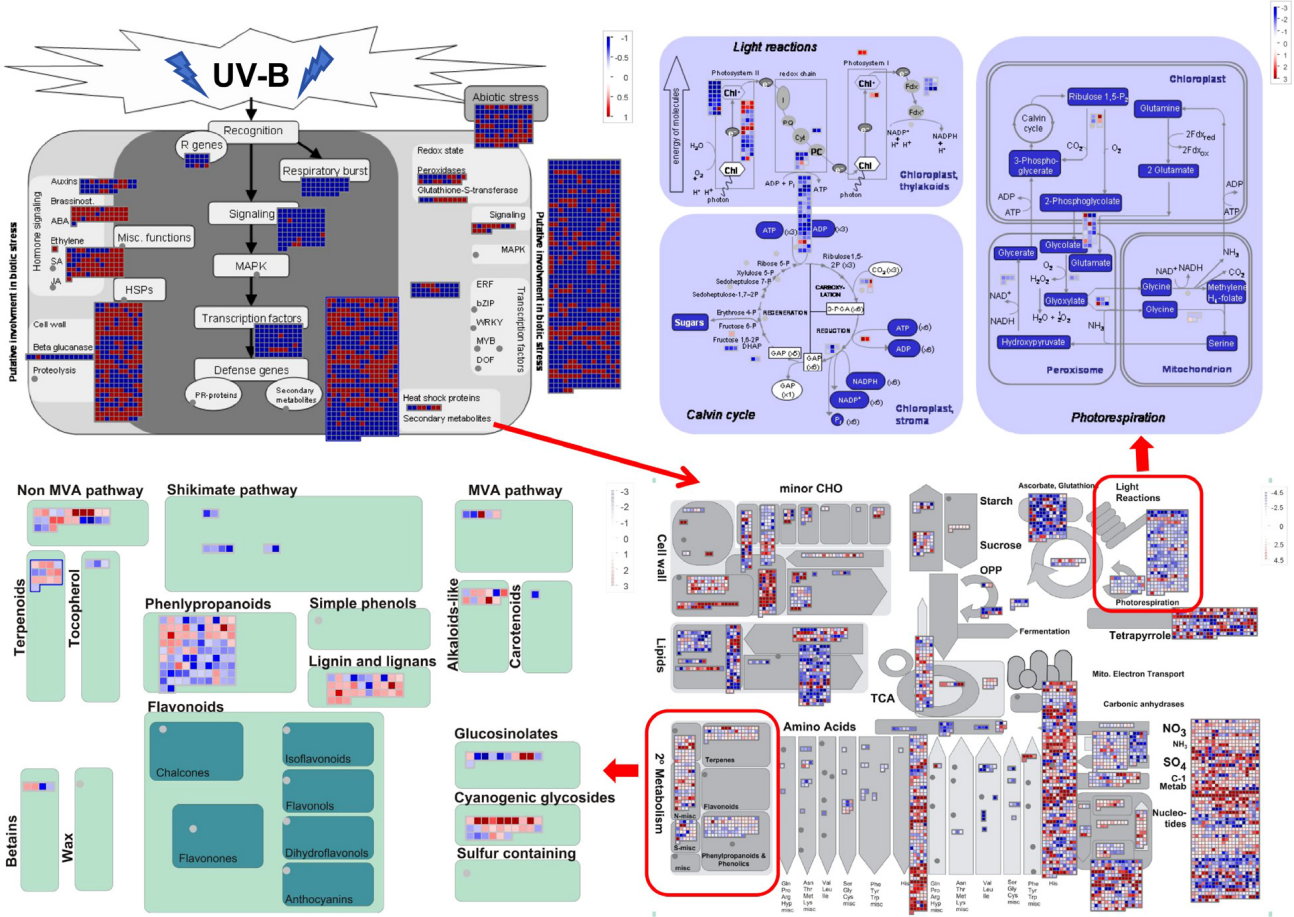
MapMan diagram of *G. hirsutum* genes involved in the response to UV-B stress. Overview of DEGs expression patterns in UV-B stress leaves relative to untreated controls. Dots show the DEGs encoding proteins related to a certain step in the defense response. Red and blue dots indicate upregulation and downregulation, respectively.

MapMan ontology analysis was employed to construct a genome-wide map of *G. hirsutum* gene expression by identifying pathways associated with defense-related signaling and metabolic responses. We conducted MapMan analysis of total 24,257 DEGs in the CK vs. UV-B comparison group. Analysis results revealed that genes involved in abiotic stress, pathogen recognition, phytohormones, cell wall metabolism, redox state, metabolic processes, and secondary metabolism (**Figure 3**). In addition, the DEGs also encoded for transcription factors (TFs) and large enzyme families.

Under UV-B stress, the expression of various genes exhibits distinct regulatory patterns. Genes related to phytohormones (e.g. brassinosteroids, ethylene, and SA), cell wall, abiotic stress, and secondary metabolites (e.g. phenylpropanoid, lignin and lignans, cyanogenic glycosides, terpenoids, and alkaloids) were upregulated under UV-B stress. In contrast, genes associated with other phytohormones (e.g. auxins), respiratory burst, and signaling pathways were downregulated (**Figure 3**). Similarly, genes encoding TFs, such as the Ethylene Response Factor (*ERF*), were also downregulated. Analysis of biotic stress pathways revealed that DEGs were mainly enriched in cell wall-related pathways, secondary metabolism, and TFs. Further analysis of the secondary metabolic pathways indicated that the DEGs were primarily enriched in the phenylpropanoid- and lignin-related pathways. The phenylpropanoid pathway serves as an upstream pathway involved in lignin and anthocyanin biosynthesis.

**Figure 4:**
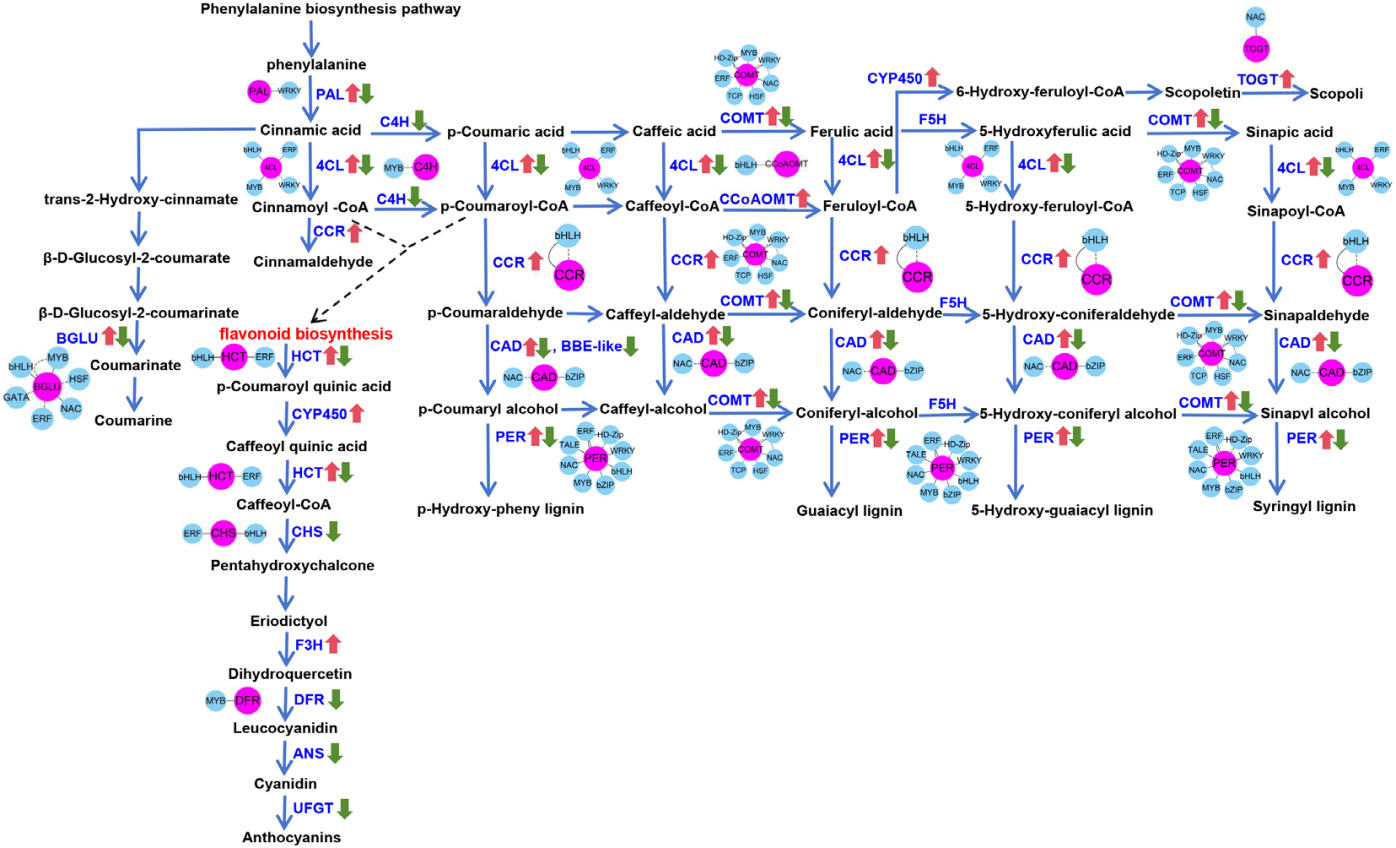
Pathway diagram of structural and regulatory genes related to the phenylpropanoid pathway. The blue words indicate the enzymes encoded by the structural genes. The red arrow indicates the upregulated genes, and the green arrow indicates the downregulated genes. Red dots represent structural genes, and blue dots represent regulatory genes.

Specifically, coumaroyl-CoA in the phenylpropanoid pathway contributes to flavonoid biosynthesis, while p-coumaryl alcohol, coniferyl-alcohol, 5-hydroxy-coniferyl alcohol, and sinapyl alcohol are involved in synthesizing various types of lignin (H-lignin, G-lignin, and S-lignin) (**Figure 4**). Similarly, a detailed analysis of the photosynthesis-related DEGs revealed that most of these genes were downregulated (**Figure 3**). Enrichment analysis indicated that UV-B stress mainly affected PSII and ATP synthesis during light reactions. These light reactions occur in the thylakoid membrane, where photosynthetic pigments absorb light energy for hydrolysis. The downregulated genes inhibit the light reaction process, thereby inhibiting the production of photosynthetic pigments. Therefore, the chlorophyll content decreased significantly after UV-B stress. MapMan analysis demonstrated that UV-B stress influenced photosynthesis in *G. hirsutum*, mainly affecting PSII and ATP production. It also alleviates the adverse effects of UV-B radiation by generating flavonoids and lignins (**Figure 3**). Here, We describe the same physiological processes from different perspectives and provide a deeper understanding of the molecular mechanisms underlying UV-B tolerance in *G. hirsutum*.

### 3.4 KEGG analysis of DEGs in *G. hirsutum* under UV-B stress

To further determine the function of DEGs, we performed KEGG enrichment analysis of DEGs. Due to KEGG functional annotations could not be found for some DEGs, these genes were removed from the KEGG enrichment analysis. Under UV-B stress, 4,689 DEGs were eventually annotated with 30 KEGG pathways. The top 20 KEGG terms were screened out based on *P*-value.

Based on the number of genes, a larger number of genes were found to be enriched in terms of MAPK signaling pathway-plant (ghi04016), starch and sucrose metabolism (ghi00500), amino sugar and nucleotide sugar metabolism (ghi00520), and phenylpropanoid biosynthesis (ghi00940), among others (**Supplementary Figure S1**). This indicates that UV-B stress affects both primary and secondary metabolism in *G. hirsutum*. The phenylpropanoid biosynthesis pathway is an upstream process in the synthesis of lignins, flavonoids, and other substances that play important roles in plant responses to abiotic stress. Consistent with the KEGG results, MapMan analysis showed that DEGs were enriched in the phenylpropanoid biosynthesis pathway of the secondary metabolic pathway. Therefore, we analyzed the phenylpropanoid pathway in detail. The DEGs related to phenylpropanoid biosynthesis were mapped using the KEGG database (**Figure 4**).

Comparative transcriptome analysis revealed that the phenylpropanoid biosynthesis pathway was enriched with a higher number of unigenes (131 DEGs), 69 genes upregulated and 62 genes downregulated (**Supplementary Table S4**). All the enzymes are encoded by different gene families, each containing more than one gene member. Gene annotation identified 81 DEGs encoding key enzymes, including 4-coumarate–CoA ligase (4CL), berberine bridge enzyme-like (BBE-like), cinnamate 4-monooxygenase (C4H), cinnamyl alcohol dehydrogenase (CAD), caffeoyl-CoA O-methyltransferase (CCoAOMT), cinnamoyl-CoA reductase (CCR), caffeic acid 3-O-methyltransferase (COMT), phenylalanine ammonia lyase (PAL), and peroxidase (PER), all of which are ultimately involved in the synthesis of various types of lignin (H-lignin, G-lignin, S-lignin) and flavonoid bioanabolic pathways (**Figure 4**). Twenty-seven DEGs were identified that encoded leucoanthocyanidin dioxygenase (ANS), chalcone synthase (CHS), Cytochrome P450 (CYP450), dihydrofavonol-4-reductase (DFR), flavanone 3-hydroxylase (F3H), shikimate O-hydroxycinnamoyltransferase (HCT), and UDP-glucose flavonoid 3-O-glucosyltransferase (UFGT), which are ultimately involved in the anthocyanin biosynthetic pathway (**Figure 4**). A total of 23 DEGs (Beta-glucosidase BGLU, scopoletin glucosyltransferase TOGT) were involved in the synthesis of the down-stream products of phenylpropanoid metabolism, including coumarin, cinnamaldehyde, and scopolin (**Figure 4**). The expression patterns of these DEGs varied, and different members of the same gene family had opposite expression patterns under UV-B stress. Among these DEGs, the genes encoding CCoAOMT, CCR, CYP450, F3H, and TOGT were upregulated; the genes encoding ANS, BBE-like, C4H, CHS, DFR, and UFGT were downregulated. But the members encoding 4CL, BGLU, CAD, COMT, HCT, PAL, and PER were both upregulated and downregulated. Changes in the expression levels of these DEGs were associated with different physiological processes. Some of these genes are involved in lignin and flavonoid bioanabolic pathways, which may lead to changes in the content of total lignin, lignin monomers, and anthocyanins. Anthocyanins were the most important flavonoids detected. We determined the anthocyanin content in *G. hirsutum* leaves and found that it increased significantly under UV-B stress. These results suggest that *G. hirsutum* responds to UV-B stress by regulating lignin and anthocyanin synthesis, leading to UV-B resistance.

To understand the regulatory networks of the phenylpropanoid metabolism pathway under UV-B stress, we employed co-expression network analysis to identify potential regulatory genes of different structural genes. Structural genes were used as targets to identify potential regulatory genes in this pathway. Finally, 40 structural genes (three *4CL*, five *BGLU*, two *C4H*, two *CAD*, one CCoAOMT, two CCR, three CHS, five *COMT*, three DFR, two HCT, one PAL, ten *PER*, and one TOGT) were ultimately found to be associated with 60 regulatory genes (13 *bHLH*, two *bZIP*, ten *ERF*, one *GATA*, six *HD-Zip*, two *HSF*, ten *MYB*, seven *NAC*, one *TALE*, two *TCP*, and six *WRKY*) (**Supplementary Table S5**). Among the regulatory genes, members of the MYB gene family were particularly prominent, with *GhMYB4* (*Gohir.A01G153200*) emerging as a key regulator that we previously predicted to regulate anthocyanin biosynthesis. Therefore, we selected a MYB family gene (*GhMYB4*, *Gohir.A01G153200*) for functional verification by *Arabidopsis* transformation.

### 3.5 Overexpression of *GhMYB4* reduced the UV-B tolerance in *Arabidopsis*

**Figure 5:**
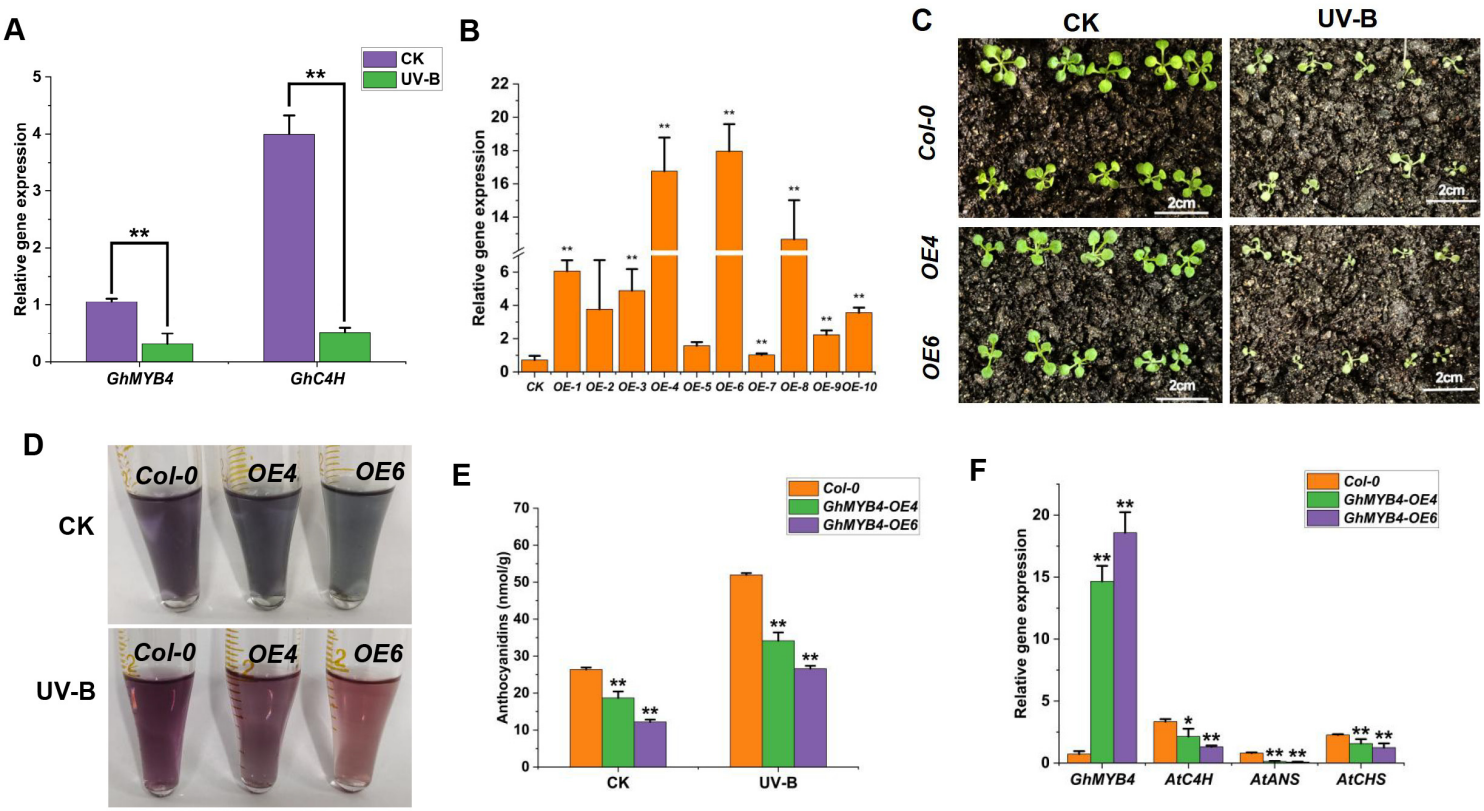
The phenotype and anthocyanins content of *Arabidopsis* transgenic overexpression lines under UV-B stress. **(A)**, The relative gene expression of *GhMYB4* and its associated gene *GhC4H* under UV-B stress. **(B)**, qRT-qPCR detection of relative expression of *GhMYB4* in WT and *GhMYB4-OE* transgenic lines. **(C)**, The growth of *Col-0, OE-4*, and *OE6* under normal growth conditions (CK) and UV-B stress. **(D, E)**, Detection of anthocyanin relative content in *Col-0, OE-4*, and *OE-6* under UV-B stress. **(F)**, The relative gene expression of *AtC4H*, *AtANS*, and *AtCHS* in overexpression lines (OE4 and OE6) compared to CK. Statistical significance was calculated using *T*-test; *P < 0.05, **P < 0.01.

Co-expression network analysis revealed that *GhMYB4* was associated with *GhC4H* (*Gohir.A13G235200*). qRT-PCR analysis revealed that the relative expression levels of *GhMYB4* and *GhC4H* significantly decreased under UV-B stress (**Figure 5A**). To further examine the role of *GhMYB4* in response to UV-B stress, we produced *Arabidopsis* lines overexpression (OE) *GhMYB4* and analyzed their physiology. We assessed the transgenic efficiency in homozygous transgenic Arabidopsis lines from the T3 generation (*OE-6*) (**Figure 5B**). The growth of *GhMYB4* overexpression lines (GhMYB4-OE4 and GhMYB4-OE6) and Col-0 plants was compared following UV-B treatment. There was almost no difference in the growth of Col-0, OE4, and OE6 plants under normal growth conditions (**Figure 5C**). However, the growth of OE4 and OE6 plants was significantly slower than that of Col-0 plants after UV-B treatment (**Figure 5C**). The anthocyanin content of OE4, OE6, and Col-0 plants after UV-B treatment was also compared (**Figure 5D**). The anthocyanin content of the GhMYB4-OE lines decreased significantly under both normal growth conditions and UV-B stress (**Figure 5E**). The relative expression levels of genes *AtC4H*, *AtANS*, *AtCHS*, and *GhMYB4* related to phenylpropanoid biosynthesis pathway were quantified. The relative gene expression of *AtC4H*, *AtANS*, and *AtCHS* was all downregulated in overexpression lines (*OE4* and *OE6*) compared to CK (**Figure 5F**). These findings suggested that *GhMYB4* reduces UV-B stress tolerance in *Arabidopsis* by negatively regulating anthocyanin synthesis.

## 4 Discussion

Under UV-B stress, the leaves of *G. hirsutum* exhibited obvious phenotypic damage and physiological responses. Plants have evolved various strategies to adapt to abiotic stress. These mechanisms involve complex physiological, biochemical, and molecular responses affecting plant growth. Therefore, a comprehensive understanding of these pathways and their interactions is crucial. Through comparative transcriptome analysis, we identified DEGs in *G. hirstium* under UV-B stress and conducted enrichment analyses and functional studies of these genes.

### 4.1 UV-B stress weakened the photosynthesis of *G. hirsutum*

**Figure 6:**
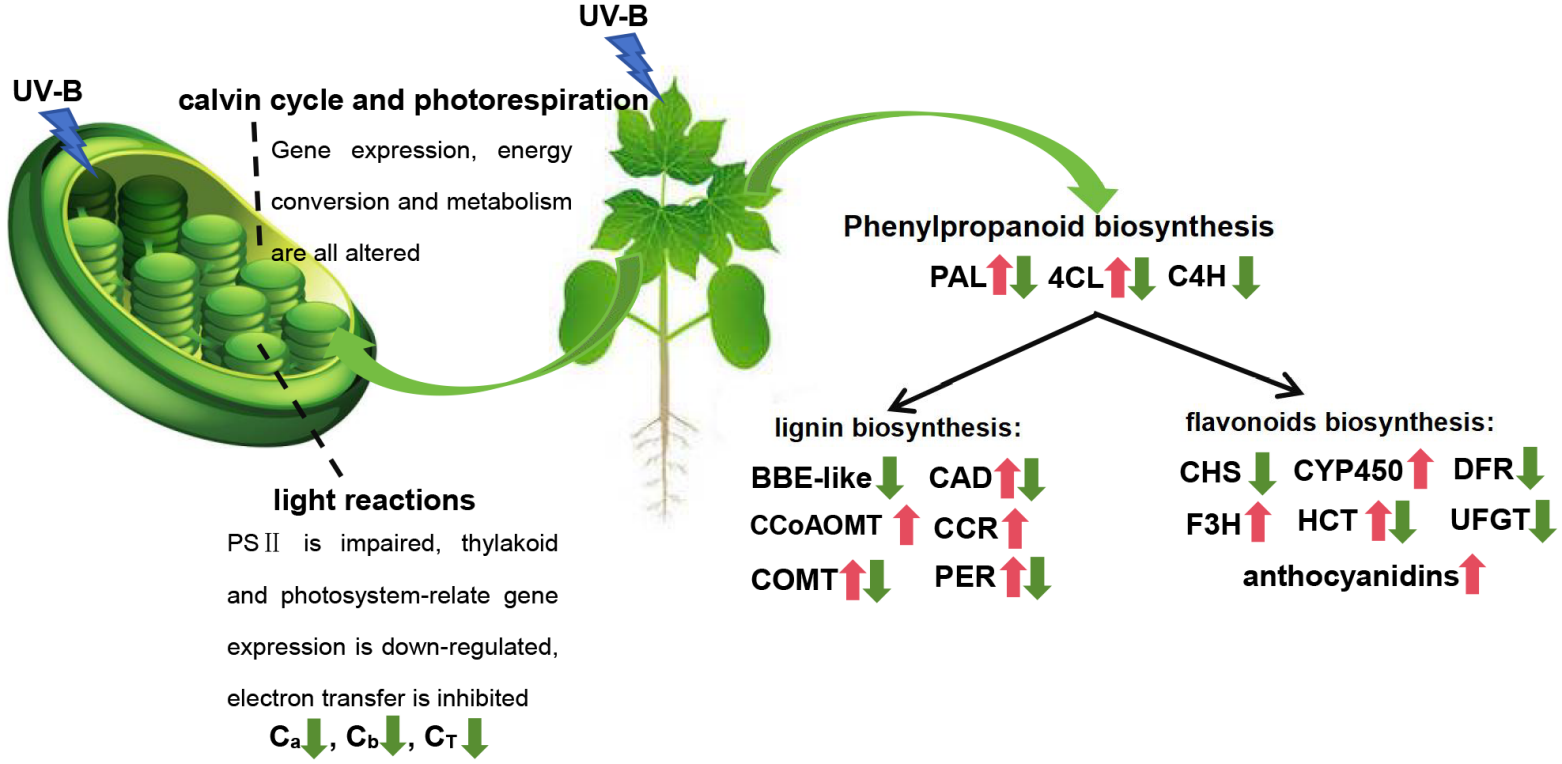
Response of *G. hirsutum* leaves to UV-B stress. The red arrow indicates upregulation and the green arrow indicates downregulation.

It was found that UV-B stress damaged the photosynthetic system, significantly decreasing the chlorophyll content of *G. hirsutum* (**Figure 6**). This phenomenon has been observed in various plants, where enhanced UV-B exposure negatively affects chlorophyll content (Rahimzadeh Karvansara and Razavi, 2019; Tripathi et al., 2021). Chloroplasts are crucial organelles involved in plant photosynthesis and environmental sensing. Photoreactions occur on the thylakoid membrane, where light-excited electrons are transferred from the reaction center to chlorophyll (Rochaix, 2011). Light-driven charge separation occurs in PSII and PSI, where electrons flow along the electron transport chain and enter the thylakoid lumen via proton pumps (Rochaix, 2011). Subsequently, a proton motive force is generated, driving ATP synthesis. UV-B stress weakens photosynthesis by damaging the PSII electron donors, inhibiting electron transfer, and reducing the efficiency of light energy utilization in photosynthetic organs (Zhou et al., 2024). Enrichment analysis revealed that UV-B stress altered the expression of genes related to photosystem and light responses. This may weaken photosynthesis by damaging PSII, inhibiting electron transfer, and reducing the efficiency of light energy utilization (**Figure 3**). The Calvin cycle plays a vital role in photosynthesis. A previous study showed that the dihydroxyacetone phosphate content in the Calvin cycle significantly increased after UV-B stress (Zhou et al., 2024). Dihydroxyacetone phosphate reacts with glyceraldehyde 3-phosphate to form D-fructose 1, 6-diphosphate. UV-B radiation also induces amino acid accumulation in *Rhododendron chrysanthum* (Zhou et al., 2024). The findings of this study are consistent with those of previous studies, showing that enhanced UV-B radiation negatively influences photosynthetic mechanisms and chlorophyll content.

### 4.2 UV-B stress affected two common metabolic branches of the phenylpropanoid pathway: flavonoids and lignin biosynthesis pathway

The levels of stress-resistant compounds, including phenylpropanoids, flavonoids, phenols, carotenoids, and anthocyanins, significantly increased under UV-B stress (Lee et al., 2021; Tripathi et al., 2021). We also found that the anthocyanin content significantly increased after UV-B treatment compared to that in the control. This finding was consistent with the that of the previous studies. To further investigate the formation and regulatory mechanisms of these stress-resistant compounds, a model was constructed using MapMan and KEGG analysis to visualize the expression of DEGs involved in defense-related signaling and metabolic pathways in *G. hirsutum* under UV-B stress. MapMan analysis revealed significant enrichment of DEGs associated with secondary metabolism. Further analysis of secondary metabolism-related pathways revealed that the DEGs were primarily enriched in phenylpropanoids, lignin and lignans (**Figure 6**). The phenylpropanoid pathway is also involved in plant stress resistance. It plays a critical role in regulating drought resistance during foxtail millet germination (Yu et al., 2020). The accumulated metabolites and DEGs associated with phenylpropanoid biosynthesis were significantly altered in tomato roots under salt stress (Jia et al., 2022). Quantification of cell morphology revealed that the cultures supplemented with phenylpropanoid-related precursors *in vitro* suffered less damage than the control group under stress (Commisso et al., 2016). Similarly in this study, DEGs associated with phenylpropanoid metabolism were enriched using MapMan secondary metabolic pathway analysis. Additionally, the phenylpropanoid biosynthesis pathway was significantly enriched in the KEGG analysis, suggesting that phenylpropanoid biosynthesis may play a typical role in the resistance to different plant stresses. The phenylpropanoid biosynthesis pathway is a precursor pathway for synthesizing various substances that produce many secondary metabolites, such as coumarin, lignin, methyleugenol, flavonoids, and anthocyanins. These secondary metabolites are also stress-resistant.

The flavonoid and lignin biosynthesis pathways, crucial in stress response, are two common metabolic branches of the phenylpropanoid pathway (Li et al., 2023a). Flavonoid and lignin biosynthesis share the same precursors and early steps (**Figure 4**). The common precursors are phenylalanine, cinnamic acid, cinnamoyl-CoA, p-coumaric acid, and p-coumaroyl-CoA (**Figure 4**). Enzymes such as 4CL, C4H, and PAL, transform and generate substances during this process. Lignin is a complex biopolymer that typically surrounds the polysaccharide components of plant cell walls (Zhong et al., 2010). As a mechanical tissue, it provides structural support and protection. Lignin biosynthesis is an important step in the phenylpropanoid metabolic pathway and plays an essential role in responding to various abiotic stresses. Various abiotic stressors, such as salt stress, drought, UV-B radiation, and low temperatures, induce changes in lignin content of several plants (Zhao et al., 2021; Li et al., 2023a, 2023b; Yang et al., 2023). In *Medicago sativa*, the phenylpropanoid pathway regulates lignin biosynthesis in response to osmotic stress (Yang et al., 2023). Similarly, the lignin and flavonoid biosynthesis pathways contribute to the response of *Morus alba* to UV-B stress (Li et al., 2023a). Changes in lignin content are attributed to variations in the levels of phenylpropanoid lignin precursors and the activities of related enzymes (Moura et al., 2010). Osmotic stress enhances lignin accumulation by increasing the guaiacyl and syringyl content, associated with alterations in the activity of enzymes involved in the metabolism of eight intermediate metabolites (Yang et al., 2023). *PoCCoAOMT* promoted lignin synthesis and ROS scavenging to resist drought stress in *P. ostia* (Zhao et al., 2021). PAL, a key enzyme in plant resistance to abiotic stress, mediates the drought tolerance in *Fritillaria unibracteata* by inducing the biosynthesis and accumulation of salicylic acid and lignin (Qin et al., 2022). Eight structural genes (*4CL*, *CAD*, *CCR*, *COMT*, *F5H*, *CYP73A*, *CCoAOMT*, and *C3′H*) were identified as significant regulators of lignin biosynthesis (Li et al., 2023b). In this study, we observed changes in the expression of various structural genes (*4CL*, *BBE-like*, *C4H*, *CAD*, *CCoAOMT*, *CCR*, *COMT*, *PAL*, and *PER*) involved in lignin synthesis, indicating that lignin biosynthesis undergoes systematic changes under stressful conditions (**Figure 6**). Genes encoding enzymes associated with secondary metabolic pathways were significantly altered, resulting in changes in compound levels.

The flavonoid biosynthetic pathway is an important branch of the phenylpropanoid metabolic pathway. Plants accumulate flavonoids in response to biological stressors (Li et al., 2023a). Flavonoids, a diverse class of phenylpropanoids, are believed to resist the penetration of UV-B radiation into sensitive leaf tissues and are involved in protecting plants from damage caused by stratospheric ozone layer depletion. Anthocyanins, a group of flavonoid pigments, are crucial for pollination by absorbing light and protecting plants from UV radiation-induced damage and cold stress (Mattioli et al., 2020). Environmental stimuli and phytohormones promote anthocyanin biosynthesis. These pigments serve as stress protectants, induced by biotic and abiotic stress conditions, and help regulate stress-induced damage. Anthocyanin content increases under high light stress to improve the tolerance in *Brassica napus* (Luo et al., 2021). Additionally, anthocyanin biosynthesis is enhanced by altering the expression levels of synthase-related genes, thereby improving cold and freezing stress tolerance in *Brassica rapa* (Ahmed et al., 2014). Similarly, the induction of anthocyanin accumulation can improve the tolerance of various plants to drought, salinity, heavy metals, nutrient limitations, and diseases (Naing and Kim, 2021; Xiang et al., 2021; Shi et al., 2023). Anthocyanin accumulation is often associated with the expression of enzymatic modification genes. Similar gene sequences from different species were compared, and their respective functions revealed a high degree of homology (Ahmed et al., 2014). In this study, we found that the expression of enzymatic modification genes (*CHS*, *CYP450*, *DFR*, *F3H*, *HCT*, and *UFGT*) related to anthocyanin biosynthesis was upregulated or downregulated under UV-B stress (**Figure 6**). These findings suggest that the anthocyanin biosynthetic pathways of different species respond similarly to various stress conditions.

### 4.3 MYB regulators play an important role in anthocyanin and flavonoid biosynthesis

Phenylpropanoids, the largest class of natural products including flavonoids, anthocyanins, lignins and tannins, perform multiple functions including biotic and abiotic stress responses (Pratyusha and Sarada, 2022). The plant-specific transcription factor *MYB* regulates the expression of structural genes involved in anthocyanin and flavonoid biosynthesis. Recent studies have revealed that *MYBs* can positively or negatively regulate anthocyanin biosynthesis. *MYB4* acts as a transcriptional repressor of key flavonoid biosynthesis genes and reduces flavonoid accumulation following UV-B exposure (Zhang et al., 2014; Banerjee et al., 2024). Additionally, it has been found that *MYB20*, *MYB14*, *MYB44* and *MYB4a* inhibit the flavonoid biosynthesis pathway in blueberry under UV-B radiation (Song et al., 2022). *LcMYBx* competes with *LcMYB1* for *LcbHLHs*, preventing the activation of *LcDFR* by *LcMYB1*-*LcbHLHs* complex, and negatively regulating anthocyanin biosynthesis (Zhao et al., 2022). *BrMYBL2.1-G* actively inhibits pigment accumulation by inhibiting the transcription of anthocyanin biosynthesis genes (Kim et al., 2022). In our previous study, 31 potential R2R3-MYB regulatory genes involved in the anthocyanin biosynthesis pathway of *G. hirsutum* were predicted using co-expression network analysis (Zhu and Bao, 2021). Our results are consistent with those of previous studies showing that *GhMYB4* directly regulates the expression of *C4H* in the phenylpropanoid and flavonoid pathways, ultimately affecting anthocyanin synthesis. However, the functions of other predicted regulatory genes require further verification.

In summary, the MapMan and KEGG enrichment analyses demonstrated that UV-B stress affected the morphology, physiology, and secondary metabolism of *G. hirsutum*. Further analysis revealed that *G. hirsutum* primarily responded to UV-B stress by affecting the phenylpropanoid metabolic pathway. This pathway played a key role in UV-B stress resistance by producing necessary precursors of lignin and flavonoids biosynthesis, ultimately leading to the synthesis of lignin and anthocyanidins, respectively. Our findings further revealed the complex physiological and metabolic changes, and intricate regulatory networks, involved in *G. hirsutum*’s response to UV-B stress. *GhMYB4* was selected for Arabidopsis transformation, and was found to respond to UV-B stress by negatively regulating anthocyanin synthesis. Further functional characterization of other candidate genes involved in the defense of *G. hirsutum* against UV-B stress is warranted. This study provides a valuable foundation for further analyses of the molecular mechanisms underlying UV-B stress resistance in *G. hirsutum* and offers insights for breeding stress-resistant *G. hirsutum* cultivars.

## Data Availability

The datasets presented in this study can be found in online repositories. The names of the repository/repositories and accession number(s) can be found in the article/**Supplementary Material**.

## Supplementary material

Supplementary Figure 1 KEGG analysis (top 20 terms) of DEGs in *G. hirsutum* under UV-B stress.
