# Supplementary figures and images for: Identification and characteristics of differentially expressed genes under UV-B stress in Gossypium hirsutum
Source: Front Plant Sci. 2025 Jan 15;15:1529912. doi: 10.3389/fpls.2024.1529912 (PMC11774880; doi:10.3389/fpls.2024.1529912)

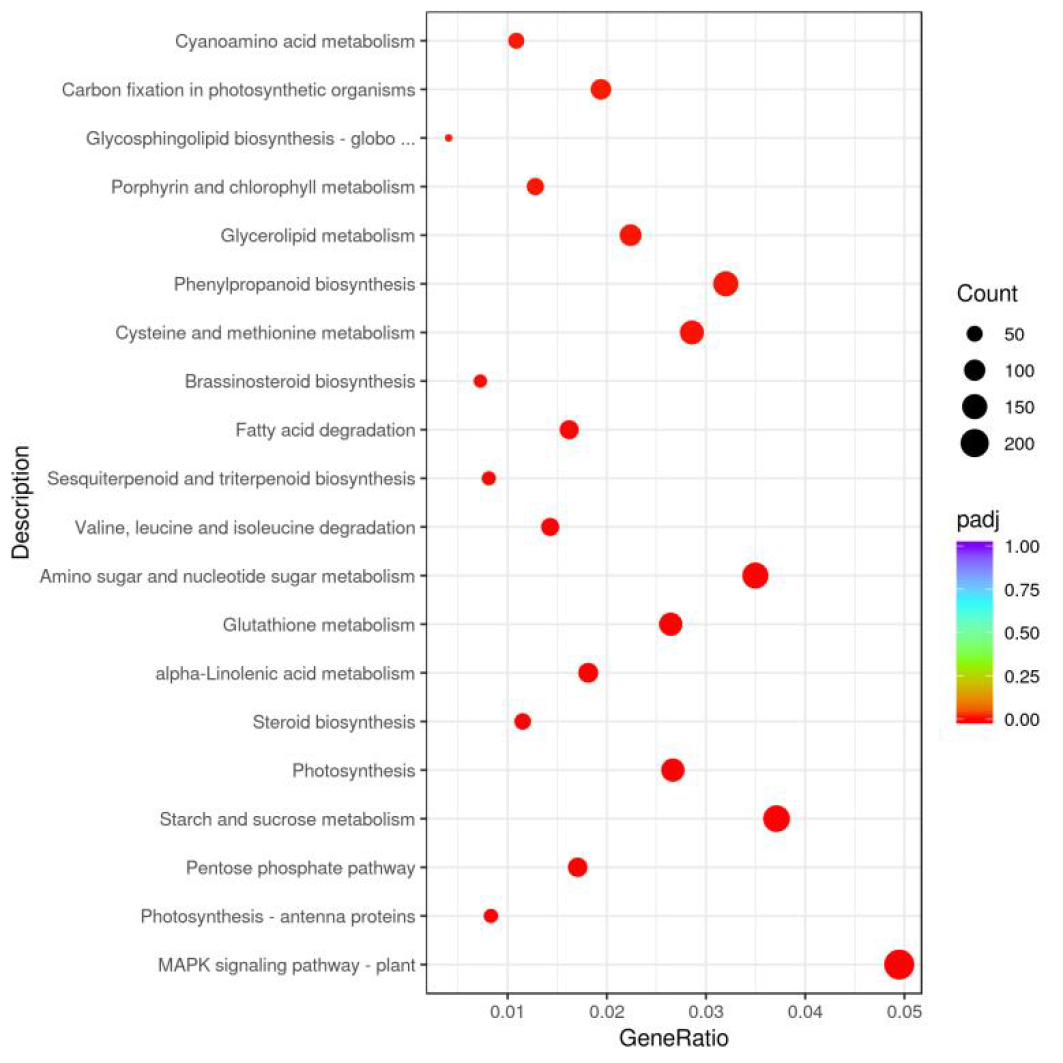

Supplement: Supplementary Figure 1 — KEGG analysis (top 20 terms) of DEGs in G. hirsutum under UV-B stress. [file Image1.tif]
